# Maternal morbidity measurement tool pilot: study protocol

**DOI:** 10.1186/s12978-016-0164-6

**Published:** 2016-06-09

**Authors:** Lale Say, Maria Barreix, Doris Chou, Özge Tunçalp, Sara Cottler, Affette McCaw-Binns, Gathari Ndirangu Gichuhi, Frank Taulo, Michelle Hindin

**Affiliations:** UNDP/UNFPA/UNICEF/WHO/World Bank Special Programme of Research, Development and Research Training in Human Reproduction (HRP), Department of Reproductive Health and Research, World Health Organization, 20 Avenue Appia, Geneva, 1211 Switzerland; Department of Community Health and Psychiatry, University of West Indies - Mona, 3 Gibraltar Camp Way, 7 Kingston, Jamaica; Jhpiego, P.O. Box 66119-00800, Nairobi, Kenya; College of Medicine, University of Malawi, P.O. Box 1131, Chipatala Avenue, Blantyre, Malawi

**Keywords:** Maternal morbidity, Definition, Measurement, Maternal health, Pilot

## Abstract

**Background:**

While it is estimated that for every maternal death, 20–30 women suffer morbidity, these estimates are not based on standardized methods and measures. Lack of an agreed-upon definition, identification criteria, standardized assessment tools, and indicators has limited valid, routine, and comparable measurements of maternal morbidity. The World Health Organization (WHO) convened the Maternal Morbidity Working Group (MMWG) to develop standardized methods to improve estimates of maternal morbidity. To date, the MMWG has developed a definition and provided input into the development of a set of measurement tools. This protocol outlines the pilot test for measuring maternal morbidity in antenatal and postnatal clinical populations using these new tools.

**Methods:**

In each setting, the tools will be piloted on approximately 250 women receiving antenatal care (ANC) (at least 28 weeks pregnant) and 250 women receiving postpartum care (PPC) (at least 6 weeks postpartum). The tools will be administered by trained health care workers. Each tool has three modules as follows:

1. personal history – socio-economic information, and risk-factors (such as violence and substance abuse)
2. patient symptoms – WHO Disability Assessment Schedule (WHODAS) 12-item, and mental health questionnaires, General Anxiety Disorder, 7-item (GAD-7) and Personal Health Questionnaire, 9-item (PHQ-9)
3. physical examination – signs, laboratory tests and results.

**Discussion:**

This pilot (planned for Jamaica, Kenya and Malawi) will allow for comparing the types of morbidities women experience between and across settings, and determine the feasibility, acceptability and utility of using a modified, streamlined tool for routine measurement and summary estimates of morbidity to inform resource allocation and service provision. As part of the post-2015 Sustainable Development Goals (SDGs) estimating and measuring maternal morbidity will be essential to ensure appropriate resources are allocated to address its impact and improve well-being.

**Electronic supplementary material:**

The online version of this article (doi:10.1186/s12978-016-0164-6) contains supplementary material, which is available to authorized users.

## Plain English summary

While there has been a lot of attention to preventing women from dying during pregnancy and childbirth, less attention has been paid to women who survive pregnancy but have health problems. We developed a tool to collect information on the kinds of health problems women may have during pregnancy. This tool includes questions on the woman’s pregnancy history; how she feels (emotionally and physically); and an examination. The tool will be tested in three countries (Jamaica, Kenya and Malawi). Approximately 1500 women, who are currently pregnant (28 weeks) or who recently had a birth (six weeks ago), will be asked to participate in testing the tool. Most questions and the examination, are part of normal care for pregnant women. We will analyse the information collected with the tool to understand the most common conditions women experience in each of the three countries, and to figure out the best ways to measure the problems women may experience related to pregnancy. We will share results of this project with the facilities where we conducted the study as well as with the health and academic communities.

## Background

Improving maternal health and reducing related mortality have been key concerns of the international community, particularly as part of the 5^th^ Millennium Development Goal (MDG-5) and now of the 3^rd^ Sustainable Development Goal (SDG-3) [1, 2]. However, maternal mortality accounts for only a small fraction of the overall burden of poor maternal health as it excludes maternal morbidity. The true extent and burden of maternal morbidity is not known. It has been suggested that for each maternal death, 20 or 30 women suffer from maternal morbidity [3, 4]. However, these calculations are not based on standardized, well-documented, or transparent methodologies. There have been significant recent advances in monitoring and improving women’s quality of care related to severe maternal morbidity, or near-miss events [5]; however accurate and routine measurements of less-severe maternal morbidity are lacking. Better measures to document and monitor maternal morbidity will help inform policy and program decisions and resource allocations to improve maternal health. This protocol describes a study aiming to develop and test a tool to measure maternal morbidity during the antenatal and postpartum periods. The tool was developed by the Maternal Morbidity Working Group (MMWG) established by World Health Organization (WHO) to improve conceptual and operational understanding of maternal morbidity.

### Defining maternal morbidity

**Fig. 1:**
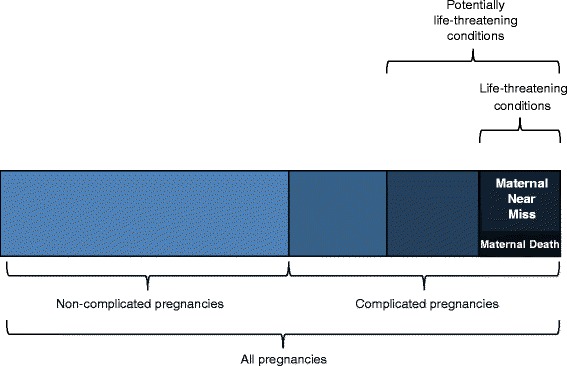
Maternal morbidity and disability spectrum [6]

The MMWG, composed of medical professionals, researchers, country programme implementers, and patient advocates, was brought together to develop a definition, identification criteria, a tool and indicators to systematically measure maternal morbidity. Figure 1 visually details the continuum of outcomes from healthy pregnancies to death [6]. The objective of the MMWG was to capture the less severe parts of the morbidity spectrum, excluding mortality and maternal near miss. The detailed methodology of the group’s work is documented elsewhere [7].

Based on a consensus process, the MMWG developed and adopted the following operational definition of maternal morbidity: “*any health condition attributed to and/or complicating pregnancy, and childbirth that has a negative impact on the woman’s wellbeing and/or functioning*” [4]. The MMWG operationalized this definition by creating a maternal morbidity matrix (Additional file 1: Table S1; Additional file 2: Table S2; Additional file 3: Table S3; Additional file 4: Table S4). The matrix was informed by literature reviews, and the tenth revision of the International Statistical Classification of Diseases and Related Health Problems (ICD-10), including the *WHO Application of ICD-10 to deaths during pregnancy, childbirth and the puerperium: ICD-Maternal Mortality* (ICD-MM) [4, 8].

### Setting the foundation for the measurement tools: operationalizing the maternal morbidity definition

The matrix highlights three dimensions of maternal morbidity which create the foundation for the measurement tools. The **first dimension** is composed of 121 conditions, 58 symptoms, 29 signs, 44 investigations and 35 management strategies. The following criteria were developed and agreed upon for inclusion in the matrix:

1. Conditions associated with a negative maternal outcome that are either exclusive to pregnancy, childbirth, or the postpartum state,
2. Conditions that occur in >0.1 % in pregnancy;
3. Conditions that are not exclusive to pregnancy, childbirth, or postpartum but which occur more frequently during pregnancy (i.e. pregnancy is a risk factor for the condition).

The identified conditions are grouped in line with the ICD-MM, with the intent of showing how data on signs, symptoms, investigations and management strategies may be aggregated together and to ensure continuity between the spectrum of morbidity through mortality [8].

The **second dimension** of the matrix measures functional impact and disability related to pregnancy, as defined in the International Classification of Functioning, Disability and Health (ICF), and is measured using the WHO Disability Assessment Schedule 2.0 (WHODAS 2.0) [9, 10]. The WHODAS covers six domains in line with ICF (cognition, mobility, self-care, getting along, life activities and participation) and produces standardized disability levels and profiles using a short, simple and easy to administer 12-item questionnaire [10].

The **third dimension** measures maternal history, focusing on social- and health-related characteristics, which might help identify the maternal morbidity as well as influence the risk and severity of the morbidity. Some examples include socio-economic status, pre-existing health conditions, and care seeking during pregnancy. These measures allow for a more comprehensive understanding of the “woman as a whole”.

### Development of maternal morbidity measurement tools

Based on the matrix, a set of tools was developed to measure maternal morbidity at two time periods - one to administer during antenatal care (ANC) and another during postpartum care (PPC). Wherever possible, previously validated scales were used such as the WHODAS 12-item for functioning, the 7-item Generalized Anxiety Disorder (GAD-7) scale and the 9-item Patient Health Questionnaire (PHQ-9) diagnostic instruments for anxiety and depression, respectively [10–12].

The study is designed to pilot the tool to:

1. determine the feasibility, acceptability and utility of implementing a modified, streamlined tool for measurement and summary estimates of morbidity to inform resource allocation and service provision
2. compare the types of morbidities women experience between and across settings.

## Methods

### Study design

The study will be cross-sectional, providing a snapshot of maternal morbidity in two study populations (ANC and PPC) in three country settings (Jamaica, Kenya and Malawi). The study will involve the administration of a questionnaire (the aforementioned maternal morbidity tool, presented in Additional files 5 and 6) at the appropriate visit where women are already coming to the facility for care.

To describe the different types of morbidity, and stratification by country setting and time of administration (ANC vs PPC), 500 women per country (250 each for ANC and PPC), were deemed adequate for capturing a range of morbidities. Without pooling the data across sites or populations, we will have a 6 % margin of error.

### Tool development and data quality

A systematic literature review was conducted to identify existing tools and scales to measure aspects of maternal morbidity. Existing measures were brought together to ensure all elements of the maternal morbidity matrix were covered. A draft version of the tool was then reviewed by the Principal Investigators (PIs) from each site, for applicability and feasibility, including the burden on participants. Mock interviews were conducted in each setting to evaluate the flow, content and timing for administering the tool. These mock interviews provided preliminary information on the questions in the tool and participant burden. In each of these steps, the questionnaire was further refined and streamlined.

The final pilot questionnaire includes three sections: 1) woman’s history, 2) current symptoms, and 3) a physical examination, including a brief review of her medical records, where available. The tools will focus on the index pregnancy and the woman’s perception of her pregnancy and health. The physical examination will include: a general overview, breast, abdominal, obstetric (for ANC patients) and pelvic (where appropriate) evaluations, in line with routine ANC and PPC examinations.

Each country pilot will be led by local investigators who will be responsible for adapting and, where appropriate, translating the questionnaires to ensure their validity and reliability in the study area.

### Enrolment, training and consent

Women attending designated facilities for routine maternal health care will be invited to participate in the study. Women for the ANC tool will be invited to participate if they are in their third trimester of pregnancy (28 or more weeks). Women for the PPC tool will be invited to participate if they are approximately 6 or more weeks postpartum. A convenience sampling strategy will be used so that all eligible women will be invited to participate until 250 women are interviewed for each tool (ANC and PPC). Data collection is anticipated to last 2 months at each site.

Local investigators will recruit, train, and supervise data collectors. Data collectors will be compensated for their participation in the research. As part of the training process in each country, teams will carefully review each question and conduct mock interviews with training participants (data collectors) who have experience in both ANC and PPC service delivery. The team will check the final version and update the consent forms as needed based on these experiences.

Training will emphasize the importance of informed consent and procedures to reduce the risk of interviewers coercing patients to participate in this study. Data collectors trained specifically for this project, will administer informed consent forms (verbal and paper based) to eligible women. Participation will be completely voluntary and non-participation will not affect a woman’s access to or the type of care due to her. This will be expressed to all potential participants during both recruitment and the informed consent session. Additionally, informed consent will ask for access to the woman’s medical records, those available at the facility and those she brings with her (mother-baby book, etc). If the woman is unable to give consent due to mental or physical impairment, she will not be asked to participate in the study. Additionally, data collectors will be trained to exclude minors under the age of 15.

The data collectors will also be responsible for referring women to appropriate services when their answers and/or physical exam deem it necessary. The local research team will identify the most appropriate places for referring women, in accordance with local standard of care. In cases where referrals will need to be outside of the facility where data collection is taking place, local PIs will contact the referral sites to confirm that the services are available prior to commencing data collection. Local supervisors will monitor and conduct random checks of interviewers to ensure informed consent and appropriate referral procedures are being followed.

The team expects that each woman’s interview will last approximately 45 to 65 min total for the administration of the tool. The physical exam should take between 15 to 25 min, while the interview portion of the questionnaire should take approximately 30 to 40 min. Information being sought on the PPC tool is more comprehensive than the routinely collected data at standard postpartum visits and participants will be informed of this during the consent process.

### Data management and statistical analysis

Data collectors will receive and be trained to use a tablet for administering the questionnaire/tool and entering the woman’s data. The tablets will support prompt data collection, transmission, verification, storage and analysis. In addition to the tablets, data collectors will have access to paper forms of the tool, as back up. All tablets will be password protected to ensure confidentiality. Project data will be inputted into electronic forms of either the ANC or PPC survey using Open Data Kit (ODK) an open source data management application on the tablets. The uploaded data will not include any identifying information on the woman, and only an ID number will be used to identify participants. Data from the tablets will be uploaded to a secure, password protected cloud-based storage system owned by WHO (https://whodcp.org). This system allows for both data entry and uploading and remote review and management of collected data.

Using tablets for administration of the tool will help ensure data quality with range checks and reduce mistakes associated with manual data entry. Real-time uploading of data to a cloud server will ensure data quality is continually monitored, by the local team and at WHO. The team based in Geneva, in conjunction with site coordinators and PIs, will be responsible for the data analysis. The process will begin while data collection is still on-going in order to assess progress and determine any data collection problems and/or patterns. Once data collection and clean-up are complete the team will perform in-depth analyses using STATA analytical software in order to synthesize and present results. In addition to the Geneva-based team, core MMWG members will be involved in interpreting the data and providing expertise when necessary.

### Ethical considerations

Ethical approval for this study was provided by the WHO’s Research Ethics Review Committee (ERC) as well as by the RHR Research Project Panel (RP2), the external review body of the Department of Reproductive Health, and Research (RHR) including the UNDP/UNFPA/WHO/World Bank Special Programme of Research, Development, and Research Training in Human Reproduction (HRP) (Additional file 7). Furthermore, relevant entities at each of the three country sites also provided approval.

There will be no risk to the women who decide not to participate in the study, they will receive the same standard of care as those who participate in the study. For women who chose to participate, this study may cause some discomfort in terms of the routine physical exams, or when answering personal questions if they are associated with negative experiences (i.e. medical and obstetric history questions about domestic violence or psychological issues). Potential benefits for participants include possible diagnosis and treatment for any reported morbidity or other condition.

Only the study team will have access to the information collected and it will remain confidential. Site coordinators will work in conjunction with data collectors to protect participant anonymity. All participants will receive a small token of appreciation for their participation.

## Discussion

Data gathered from this effort will provide better information as to the breadth and depth of pregnancy-related morbidity and disability in the three study settings. By identifying current gaps in the care of pregnant women, this study can enable researchers, policymakers and health professionals to inform program and resource planning to address women’s reproductive health needs. This study will pilot and assess the feasibility of employing a tool to measure the health consequences of pregnancy. This pilot study is a step towards finding such a tool and will provide evidence for the first standard global definition and classification of non-severe maternal morbidity. Ultimately, the goal of this project is to produce a valid, comparable, and routine tool for measurement of maternal morbidity.

### Plans for dissemination and use of project results

When the data analysis is complete, the results will be disseminated in pilot study countries, as well as through scientific journal articles. Furthermore, according to the findings, the tool will be revised, simplified and finalized as a standard measurement for monitoring maternal morbidity in country programmes.

## Conclusion

This paper describes a study designed to test a tool measuring the impact pregnancy and childbirth have on the health of women. We describe the design of the study, the tool, and how we will invite women to participate in the study. Also, we discussed ethical issues, including that even if women refuse to participate, they will still receive the same care at the facility. Our objective in conducting this study is find out the health conditions women may experience in the three countries. Based on this study, we will make changes to the tool so that it can be used to improve the health care of pregnant women and those who have recently given birth.

## Additional files

Additional file 1: Table S1. Dimension 1: Symptom, Sign, Investigations & Management (Direct Maternal Morbidity). (DOCX 21 kb) Additional file 2: Table S2. Dimension 1: Symptom, Sign, Investigations & Management (Indirect Maternal Morbidity). (DOCX 24.6 kb) Additional file 3: Table S3. Dimension 2: FUNCTIONAL IMPACT – International Classification for Functioning and Disability (ICF) Codes. (DOCX 20.3 kb) Additional file 4: Table S4. Dimension 3: MATERNAL HISTORY. (DOCX 18.6 kb) Additional file 5: Maternal morbidity pilot measurement tool—ANC. (DOC 1.20 mb) Additional file 6: Maternal morbidity pilot measurement tool—PPC. (DOC 873 kb) Additional file 7: WHO—ERC Approval. (DOC 563 kb)

## Abbreviations

ANC: antenatal care
ERC: Research Ethics Review Committee
GAD-7: General Anxiety Disorder - 7-item version
HRP: UNDP/UNFPA/WHO/World Bank Special Programme of Research, Development, and Research Training in Human Reproduction
ICD: International Statistical Classification of Diseases and related health problems
ICD-MM: WHO Application of ICD-10 to deaths during pregnancy, childbirth and the puerperium: ICD-Maternal Mortality
ICF: International Classification of Functioning, Disability and Health
MDGs: Millennium Development Goals
MMWG: Maternal Morbidity Working Group
MNM: maternal near-miss
ODK: Open Data Kit
PHQ-9: Personal Health Questionnaire- 9-item version
PPC: postpartum care
RHR: Reproductive Health and Research Department
RP2: RHR Research Project Panel
SDGs: Sustainable Development Goals
WHO: World Health Organization
WHODAS 2.0: WHO Disability Assessment Schedule 2.0
