# Supplementary material for: Maternal morbidity measurement tool pilot: study protocol
Source: Reprod Health. 2016 Jun 9;13:69. doi: 10.1186/s12978-016-0164-6 (PMC4899915; doi:10.1186/s12978-016-0164-6)
Supplement: Additional file 1: Table S1. — Dimension 1: Symptom, Sign, Investigations & Management (Direct Maternal Morbidity). (DOCX 21 kb) [file 12978_2016_164_MOESM1_ESM.docx]

**Additional file 1: Table S1 - Dimension 1: SYMPTOM, SIGN, INVESTIGATIONS & MANAGEMENT (Direct Maternal Morbidity)**

|  | **Symptom** | **Sign** | **Investigations** | **Management** |
| --- | --- | --- | --- | --- |
| **DIRECT MATERNAL MORBIDITY** | | | | |
| **Delivery/Termination** | | | | |
| Ectopic Pregnancy  Unsafe Induced Septic Abortion  Retained Products of Conception  Gestational Trophoblastic Disease  Obstructed Labour | abdominal/pelvic pain  vaginal bleeding  fever  labour > 12 hours | changes in heart rate  changes in blood pressure  fever  abnormal pelvic exam  products of conception on pelvic exam | beta hcg  pelvic/transvaginal ultrasound | methotrexate  surgery  antibiotics  chemotherapy |
| **Hypertensive Disorders of Pregnancy** | | | | |
| Hypertensive Disorders  (Chronic hypertension, Gestational hypertension, Pre-eclampsia, HELLP, Eclampsia) | headache  visual disturbance  chest pain  nausea and vomiting  abdominal pain  seizures | changes in blood pressure (hypertension)  abnormal cardiac exam  abnormal fundoscopy exam  abnormal respiratory exam  abnormal abdominal exam | uric acid  creatinine  urine analysis  complete blood count  blood smear  liver enzymes  CT scan  chest X ray  urine protein to creatinine ratio | antihypertensives  magnesium sulphate  diuretics  antenatal corticosteroids  blood transfusion |
| **Obstetric Haemorrhage** | | | | |
| Accreta/Increta/Percreta Placenta (Morbidly adherent placenta)  Placenta Previa  Placental Abruption  Postpartum Haemorrhage | abdominal pain  back pain  vaginal bleeding  uterine contractions | changes in heart rate  changes in blood pressure  abnormal abdominal exam | complete blood count  coagulation studies  pelvic/transvaginal ultrasound | blood transfusion  fluids  uterotonics  anti-shock garments  surgical management |
| **Pregnancy related Infection** | | | | |
| Mastitis/Breast Abscess  Chorioamnionitis  Puerperal Sepsis  UTI/Pyelonephritis  Tetanus | fever  diaphoresis  breast redness/tenderness/mass  vaginal discharge  abnormal urination  lock jaw  stiff neck  rigidity  muscle spasms | fever  changes in blood pressure (hypotension, hypertension)  changes in heart rate (tachycardia)  breast redness, firmness and pain  abnormal abdominal exam (abdominal/uterine/ suprapubic tenderness) | blood cultures  urine analysis and culture  complete blood count  breast ultrasound | fluids  antibiotics  benzodiazepines  magnesium sulphate |
| **Other Obstetric Complications** | | | | |
| **Thrombotic**  Deep Vein Thrombosis  Thrombophlebitis  Septic Ovarian/Pelvic Vein Thrombophlebitis  **Gastrointestinal**  Nausea and Vomiting of Pregnancy  Cholestasis of Pregnancy  **Cardiovascular**  Peripartum Cardiomyopathy  Mirror Syndrome  **Endocrine**  Diabetes Mellitus (Gestational Diabetes)  Postpartum Thyroiditis  **Other**  Intentional Self Harm | unilateral leg swelling  calf tenderness  fever  abdominal pain  nausea and vomiting  pruritus  changes in breathing  oedema/anasarca  decreased exercise tolerance/fatigue  headache  visual disturbance  chest pain  abnormal urination  polydipsia  tremor  palpitations  anxiety/irritability  changes in weight  suicidal ideation | fever  changes in heart rate  changes in respiration  decreased oxygen saturation  abnormal volume exam  abnormal cardiac exam  abnormal respiratory exam  abnormal abdominal exam  abnormal fundoscopy exam  abnormal thyroid exam  tremor  unilateral leg swelling  calf tenderness  oedema/anasarca  evidence of self-harm | complete blood count  blood smear  liver enzymes  uric acid  creatinine  urine analysis  urine protein to creatinine ratio  chest X ray  CT scan  electrolytes  echocardiogram  thyroid studies  fasting blood glucose HbA1c  oral glucose tolerance test  pelvic/transvaginal ultrasound  doppler ultrasound of legs | antibiotics  anticoagulation  antiemetics  antihypertensives  diuretics  insulin  thyroid medications  fluids |
| **Unanticipated Complications** | | | | |
| **Complications related to Anaesthesia**  Aspiration Pneumonitis  Cerebral Anoxia  **Complications of Management**  *Spontaneous Vaginal Delivery*  Perineal Tear (3rd or 4th degree)  *Episiotomy*  Episiotomy infection  *Instrumental Delivery*  Vaginal wall/perineal laceration  Urethral tear/damage  Vulval Haematoma  *Caesarean Section*  Uterine perforation  Postpartum Inversion of Uterus  Caesarean Section Wound Infection  Post-operative ileus/bowel obstruction  *Other*  Ovarian hyperstimulation syndrome  Nosocomial or hospital acquired infection (UTI, C diff, pneumonia) | changes in weight  fever  altered mental status  seizures  changes in breathing  cough  abdominal discomfort, pain and/or distension  nausea and vomiting  changes in bowel habits  abnormal urination  perineal pain/rectal pressure  dyspareunia  vaginal bleeding | fever  changes in blood pressure  changes in heart rate  changes in respiration  decreased oxygen saturation  abnormal respiratory exam  abnormal neurologic exam  abnormal abdominal exam  caesarean wound redness and/or discharge  abnormal pelvic exam | complete blood count  electrolytes  creatinine  coagulation studies  ECG  chest x-ray  CT scan  vaginal swabs | surgery  fluids  blood transfusion  antibiotics  antivenom |
