# Supplementary material for: Maternal morbidity measurement tool pilot: study protocol
Source: Reprod Health. 2016 Jun 9;13:69. doi: 10.1186/s12978-016-0164-6 (PMC4899915; doi:10.1186/s12978-016-0164-6)
Supplement: Additional file 2: Table S2. — Dimension 1: Symptom, Sign, Investigations & Management (Indirect Maternal Morbidity). (DOCX 24.6 kb) [file 12978_2016_164_MOESM2_ESM.docx]

**Additional file 2: Table S2 - Dimension 1: SYMPTOM, SIGN, INVESTIGATIONS & MANAGEMENT (Indirect Maternal Morbidity)**

|  | **Symptom** | **Sign** | **Investigations** | **Management** |
| --- | --- | --- | --- | --- |
| **INDIRECT MATERNAL MORBIDITY** | | | | |
| ** See list below* | fever  chills  diaphoresis/night sweats  fatigue  changes in weight  headache  visual disturbance  cough  changes in breathing  chest pain  palpitations  light headedness  jaundice  change in appetite  nausea and vomiting  abdominal pain  changes in bowel habits  abnormal urination  vaginal bleeding  vaginal discharge  anxiety  irritability  guilt  changes in mood  lack of interest  difficulty concentrating  hallucinations  delusions  impulsiveness  change in eating habits  excessive exercise  compulsive behaviour  suicidal ideation  unilateral leg swelling and/or redness  oedema/anasarca  leg pain  back pain  joint pain  skin rash/lesion  neurological symptoms | changes in blood pressure  fever  changes in heart rate  changes in respiration  abnormal oxygen saturation  bruises  pallor  abnormal fundoscopy  abnormal mental status  lymphadenopathy  thrush (oral)  abnormal thyroid exam  abnormal cardiac exam  abnormal respiratory exam  abnormal abdominal exam  abnormal neurological exam  abnormal rectal exam  abnormal pelvic exam  skin rash/lesion  abnormal musculoskeletal exam | complete blood count  blood smear  thick and thin smear for malaria  haemoglobin  electrophoresis  fasting blood glucose  HbA1c  oral glucose tolerance test  electrolytes  creatinine  urine analysis and urine protein: creatinine ratio  chlamydia/gonorrhea PCR/ cultures  viral PCR/ culture  vaginal swab  influenza swab  hepatitis serology  AFB stain and culture  stool culture  iron studies  vitamin B12 level  thyroid studies  PTH level  calcium  ECG  echocardiogram  pulmonary function tests  overnight oximetry  skin biopsy  bone marrow biopsy  CT scan  thoracentesis  paracentesis | antibiotics  antihypertensives  insulin  oral hypoglycemics  anti retrovirals  anti-malaria drugs  TB medications  anti virals  anti fungals  vitamin and mineral supplementation  thyroid medications  anti-psychotics  anti-depressants  diuretics  other cardiac medications [beta blockers, ace inhibitors]  steroids  immunosuppressants  chemotherapy  radiation  surgery |
| **CO-INCIDENTAL** | | | | |
| **External injury in pregnancy**  Motor Vehicle Accident (Transport accidents)  Accidental exposure to smoke, fire, flames  Accidental poisoning and exposure to noxious substance  Accidental drowning and submersion  Contact with venomous animals and plants  Exposure to force of nature  **Trauma in pregnancy**  Falls  Intimate Partner Violence  Rape | history of external injury and/or trauma  bleeding  pain  difficulty with mobility  changes in breathing  altered mental status  seizures  vaginal bleeding  dyspareunia | changes in blood pressure hypothermia  decreased oxygen saturation  fractures  burns  bruises  abnormal respiratory exam  abnormal neurologic exam  abnormal musculoskeletal exam | complete blood count  electrolytes  creatinine  coagulation studies  ECG  chest x-ray  CT scan  vaginal swabs | surgery  fluids  blood transfusion  antibiotics  antivenom |

***INDIRECT MATERNAL MORBIDITY- CONDITIONS**

Pre-existing Hypertension

Pre-exiting Diabetes Mellitus

**Maternal infectious and parasitic diseases classified elsewhere but complicating pregnancy, childbirth and the puerperium**

HIV/AIDS

Tuberculosis Mycobacterium

Malaria

*Sexually Transmitted Infections*

Chlamydia

Anogenital warts

Herpes Simplex

Syphilis

*Other*

Candidiasis

Influenza

Pneumonia

Infectious Hepatitis (A, B, C, E)

Varicella Zoster

Cholera

**Other Maternal Diseases Classifiable Elsewhere but Complicating Pregnancy, Childbirth and the Puerperium**

*Acquired* *Anemia*

Anemia due to vitamin B12 and/or folate deficiency

Iron Deficiency Anemia

*Hereditary Anemia*

Sickle Cell Anemia

Thalassemia

**Other diseases in the blood and blood forming organs and certain disorders involving the immune mechanism complicating pregnancy, childbirth and the puerperium**

Idiopathic Immune Thrombocytopenia (ITP)

**Endocrine, nutritional and metabolic diseases complicating pregnancy, childbirth and the puerperium**

Hyperparathyroidism

*Thyroid disorders*

**Mental disorders and diseases of the nervous system complicating pregnancy, childbirth and the puerperium**

*Anxiety Disorders*

Adjustment Disorder

Anxiety Disorder

Panic Disorder

Post-traumatic Stress Disorder

Tocophobia (specific isolated phobias)

*Mood Disorders*

Bipolar Disorder

Major Depressive Disorder

Postpartum Blues

Postpartum Depression

Psychosis

Puerperal Psychosis

Schizophrenia

**Diseases of the circulatory system complicating pregnancy, childbirth and the puerperium**

Acquired and Congenital Structural Heart Disease (including valvular heart disease)

Aortic Dissection

Arrhythmia

Cardiomyopathy (dilated and restrictive)

**Diseases of the respiratory system complicating pregnancy, childbirth and the puerperium**

Asthma

Obstructive Sleep Apnea

Pulmonary Embolism

**Diseases of the digestive system complicating pregnancy, childbirth and the puerperium**

Anal Fissure

Hemorrhoids

Cholecystitis/cholylethiasis

Gastrointestinal Esophageal Reflux Disease (GERD)

Inflammatory Bowel Disease

**Diseases of the Genitourinary System**

Acute and Chronic Kidney Disease

Incontinence (urge, stress)

Uterine/Uterovaginal Prolapse (including cystocele)

Recto-vaginal fistula

Vesico-vaginal fistula

**Diseases of the skin and subcutaneous tissue complicating pregnancy, childbirth and the puerperium**

*Pregnancy Specific Dermatoses*

Eczema (Atopic dermatitis)

Prurigo of Pregnancy (Diseases of the skin and subcutaneous tissue complicating pregnancy, childbirth and the puerperium)

Pruritic Urticarial Papules and Plaques of Pregnancy (PUPP) or Polymorphic Eruption of Pregnancy (PEP) Linea nigra

*Dermatoses aggravated by pregnancy*

Acne

Psoriasis

**Other specified diseases and conditions complicating pregnancy, childbirth and the puerperium**

**Diseases of the Nervous System**

Bell's Palsy

Carpal Tunnel

Migraine

Multiple Sclerosis

Restless Leg Syndrome

Seizure disorder (excluding eclampsia)

**Diseases of the Muskoskeletal System and Connective Tissue**

Inflammatory arthritis

Ankylosing spondylitis

Rheumatoid Arthritis

Systemic Lupus Erythematosus (SLE)

Non-inflammatory arthritis

Back pain

**Oncology**

Cervical Dysplasia/Neoplasia

Lymphoma

Leukemia

Melanoma

**Nutritional**

Anorexia Nervosa

Bulimia Nervosa

Hyperthyroidism
