# Supplementary material for: Maternal morbidity measurement tool pilot: study protocol
Source: Reprod Health. 2016 Jun 9;13:69. doi: 10.1186/s12978-016-0164-6 (PMC4899915; doi:10.1186/s12978-016-0164-6)
Supplement: Additional file 3: Table S3. — Dimension 2: FUNCTIONAL IMPACT – International Classification for Functioning and Disability (ICF) Codes. (DOCX 20.3 kb) [file 12978_2016_164_MOESM3_ESM.docx]

**Additional file 3: Table S3 - Dimension 2: FUNCTIONAL IMPACT – International Classification for Functioning and Disability (ICF) Codes**

| Understanding | Watching (d110) |
| --- | --- |
|  | Listening (d115) |
|  | Learning (d130-d155) |
|  | Focusing attention (d160) |
|  | Reading (d166) |
|  | Writing (d170) |
|  | Calculating (d172) |
|  | Solving problems (d175) |
|  | Other specified |
| Communication | Communicating with others (d310 d315 d320 d325) |
|  | Speaking (d330) |
|  | Starting a conversation (d3500) |
|  | Sustaining a conversation (d3501) |
| Mobility | Standing (d4104) |
|  | Bending (d4105) |
|  | Maintaining a body position (d4154) |
|  | Transferring oneself (d420) |
|  | Lifting and carrying objects (d430) |
|  | Fine hand use (d440) |
|  | Hand and arm use (d445) |
|  | Walking short distances (d4500) |
|  | Walking long distances (d4501) |
|  | Vigorous activities (d455 d4303) |
|  | Moving around within home (d4600) |
|  | Moving around outside the home and other buildings (d4602) |
|  | Using transportation (d470) |
|  | Driving (d475) |
| Self-Care | Washing oneself (d510) |
|  | Caring for body parts (d520) |
|  | Urination (d5300) |
|  | Defecation (d5301) |
|  | Dressing (d540) |
|  | Eating (d550) |
|  | Drinking (d560) |
|  | Managing one's health (needs, assistance or oversight) (d570) |
| Interpersonal Relations | Making friends (d7200 d7500) |
|  | Engaging with other people (d740 d750) |
|  | Maintaining family relationships (d760) |
|  | Dealing with strangers (d730) |
|  | Engaging in sexual relationships (d7702) |
| Life Activities Household | Shopping (d620) |
|  | Cooking /preparing meals (d630) |
|  | Doing housework (d640) |
|  | Looking after/helping others |
| School | Attending school (d820) |
|  | Learning a job (vocational training, apprenticeship) (d825) |
|  | Going to university (d830) |
| Work and economic life | Engaging in paid work (d850) |
|  | Seeking employment (d8450) |
|  | Performing job related tasks (d8451) |
|  | Handling money (d860) |
| Life management | Undertaking a single task (d210) |
|  | Undertaking multiple tasks (d220) |
|  | Carrying out daily routine (d230) |
|  | Handling stress and psychological demands (d240) |
| Social Participation | Taking part in social life (d910) |
|  | Sports (d9201) |
|  | Travel (d920) |
|  | Visiting friends (d9205) |
|  | Human rights (e.g. self-determination, equal opportunities) (d940) |
|  | Political life and citizenship (e.g. voting) (d950) |
| Children and Youth | Learning to read (d140) |
|  | Learning to write (d145) |
|  | Learning to calculate (d150) |
|  | Communicating with others (d310 d315 d320 d325) |
|  | Speaking (d335) |
|  | Attending school (d8201) |
|  | Taking exams (d8202) |
|  | Playing with others (d880 d9200) |
