# Supplementary material for: Maternal morbidity measurement tool pilot: study protocol
Source: Reprod Health. 2016 Jun 9;13:69. doi: 10.1186/s12978-016-0164-6 (PMC4899915; doi:10.1186/s12978-016-0164-6)
Supplement: Additional file 4: Table S4. — Dimension 3: MATERNAL HISTORY. (DOCX 18.6 kb) [file 12978_2016_164_MOESM4_ESM.docx]

**Additional file 4: Table S4 - Dimension 3: MATERNAL HISTORY**

**Maternal Demographic Characteristics**

Age

Parity

Body Weight (pre-pregnancy weight)

**Past Obstetric History**

Recurrent miscarriage

Sub-fertility

Sexually transmitted infections

Pre-term delivery

Post-term delivery

C section

Instrumental Delivery

**Past Medical History**

HIV status

Previously diagnosed medical conditions

Past hospital visits or admissions for a medical condition

Chronic medications

**Social History**

FGM

Alcohol

Smoking

Substance Abuse

Violence

***Socio-economic determinants***

Marital status

Employment (migrant worker captured)

Education

**Care Seeking in this Pregnancy**

Number of ANC visits

Location of ANC visits

Visits other than for ANC

-Related to maternal concerns

-Related to fetal concerns

Referral to higher level facility

**History during Index Pregnancy**

IVF

Multiple gestation

Excessive weight gain in pregnancy

Low weight gain in pregnancy

Malnutrition in pregnancy

Cervical incompetence

Premature Rupture of Membranes (including PPROM)

Prolonged Labor

Threatened pre-term labor

Oligohydramnios

Polyhydramnios

Pre-term delivery

Post-term delivery

Spontaneous abortion

Threatened abortion

Missed abortion

Induced abortion

C-section

Instrumental Delivery

**Fetal Outcomes**

Congenital blindness

Fetal anomaly

Birth weight

Small baby

Large baby

Stillbirth
