# Supplementary material for: Maternal morbidity measurement tool pilot: study protocol
Source: Reprod Health. 2016 Jun 9;13:69. doi: 10.1186/s12978-016-0164-6 (PMC4899915; doi:10.1186/s12978-016-0164-6)
Supplement: Additional file 5: — Maternal morbidity pilot measurement tool—ANC. (DOC 1.20 mb) [file 12978_2016_164_MOESM5_ESM.doc]

Additional file 5: MATERNAL MORBIDITY PILOT MEASUREMENT TOOL - ANC

|  | **ANC SECTION 1: PATIENT HISTORY** | |
| --- | --- | --- |
|  | **Today's date** (yyyy/mm/dd) |  |
| Q1 | **Interviewer Name: ______________________________** | |
|  | **Informed Consent.** *Please read the attached consent form to the patient. If the patient agrees to participate have her sign or fingerprint the form and take a picture of the signature/fingerprint with your tablet for documentation purposes. If the patient declines to participate, please attempt to ask her Q5-14.* | |
| Q2 | **Patient ID Number.** *ID number is located at the top right hand corner of the attached consent form.* | **#_______________________** |
|  | **Social & Demographic Information.** *Please read the following: "I would like to start by asking you some general questions about your life. If you don't understand a question or would like me to repeat it please feel free to stop and ask me."* | |
| Q3 | In what month and year were you born? *If unknown, please enter Jan 1950* | month: ________________ year:______________ |
| Q4 | How old were you on your last birthday? |  |
| Q5 | What is the highest level of school you attended? | □ none □ primary □ secondary □ higher |
| Q6 | What is your current marital status? *Please select one of the following choices*:  *(If single, ask: ever married?)* | Never married/Single _______ Currently married _______ Separated _______ Divorced _______ Widowed _______ Cohabiting _______  Other *(please specify)* ________ |
| Q7 | Have you worked in the last 12 months? | _______ No ________Yes |
| Q8 | Were you paid for this work? | _______ No ________Yes |
| Q9 | What district/county/parish/subnational level are you staying in? | Answers vary per site |
| Q10 | How long did it take you to get from your house to your health facility today? | □ <15 mins □ 15-30 mins □ 30 mins - 1hr □ >1hr |
| Q11 | Now I would like you to read this sentence to me*:*   "The child is reading a book."  IF RESPONDENT CANNOT READ WHOLE SENTENCE, PROBE:  Can you read any part of the sentence to me? | ____ cannot read at all ____ able to read only parts of the sentence ____ able to read whole sentence ____ no card with required language ____ blind/visually impaired ____ other (please specify): ________________________ |

|  | **Obstetric History.** *Please read the following: "We are working on a project to get a better idea of how women feel throughout and after their pregnancies in order to improve what we know and how we can better serve you and other pregnant women in the future. Now I would like to ask you some questions about other times you have been pregnant, if any. Again, please ask me if you don't understand the question."* | | | | |
| --- | --- | --- | --- | --- | --- |
| Q12 | How many babies have you given birth to (that lived or died) after 28 weeks or 7 months of pregnancy? | | | |  |
| Q13 | How many babies did you lose before 28 weeks or 7 months of pregnancy? | | | |  |
| Q14 | How many children have you given birth to that are now alive? | | | |  |
| Q15 | How many times have you been pregnant? (including this pregnancy, and times when you did not give birth) | | | |  |
| A-1 | Are you currently pregnant with more than one baby? | | | | _______No _______Yes □ Don't know |
|  | **Most Recent Pregnancy/Delivery.** *Please read the following: "Now I would like to ask you some more intimate questions, if you do not feel comfortable answering them please let me know at any time."* | | | | |
| Q16 | Since you became pregnant, are you satisfied with your sex life? | _______ No ________Yes (Please skip to Q19) | | | |
| Q17 | Since you became pregnant, the problem(s) with your sex life is: *(Please select all the choices that apply)* | 1) Problem with little or no interest in sex ______ 2) Problem with decreased genital sensation (feeling) _______ 3) Problem with decreased vaginal lubrication (dryness) ______ 4) Problem reaching orgasm ______ 5) Problem with pain during sex _______ 6) Other (please specify): _______________  7) Refused to answer | | | |
| Q18 | *If more than one option is chosen for Q17, then please ask the patient* "Which problem is the most bothersome?" *and circle the corresponding answer.* | | | | |
|  | **Risk factors/Environment.** *Please read the following: "The next few questions I will ask may be a bit difficult, so please feel free to ask for a break or stop at any time. I want to remind you that this is confidential and no one will know how you answered. Also, if after this section you'd like to talk more about the questions, I will give you information on where to seek help. We are asking these questions to better understand your health situation and those of other women who might have similar experiences in the future." If the patient does not want to answer, you can skip the remaining questions and offer her access to the services available (see note at the end of the page).* | | | | |
| Q19 | During this pregnancy, have you used any of the following substances: tobacco products, alcoholic beverages, cannabis, inhalants for non-medical use? | | | _______No _______Yes | |
| Q20 | During this pregnancy, have you used any substances: sedatives or sleeping pills, hallucinogens, opioids, and/or any drugs by injection, etc., for non-medical use? | | | _______No (& No on Q19, Please skip to Q25)  _______Yes | |
| Q21 | During this pregnancy, have you failed to do what was normally expected of you because of your consumption of any of the abovementioned substances? | | | _______No _______Yes | |
| Q22 | During this pregnancy, has your use of any of the aforementioned substances led to health, social, legal, or financial problems? | | | _______No _______Yes | |
| Q23 | During this pregnancy, has a friend or relative or anyone else *ever* expressed concern about your use of any substance? | | | _______No _______Yes | |
| Q24 | During this pregnancy, have you ever tried to cut down on using any substance, but failed? | | | _______No _______Yes | |
|  | **If woman answers yes to Q19 or to Q20, please [Insert specific local instructions for health worker on how to handle case of drug abuse and referral here.]** | | | | |
|  | **Violence.** *Please read the following: "The next few questions I will ask may also be a bit difficult, so please feel free to ask for a break or stop at any time. I want to remind you that this is confidential and no one will know how you answered. Also, if after this section you'd like to talk more about the questions, I will give you information on where to seek help. We are asking these questions to better understand your health situation and those of other women who might have similar experiences in the future."* | | | | |
| Q25 | Are you afraid of your current/most recent husband or partner or anyone else? Would you say never, sometimes, many times, most/all of the time? | | _______Never _______Sometimes _______ Many times _______ Most/all of the times _______ Don't know/Don't remember _______ Refused/No answer (Skip to instructions) | | |
| Q26 | During this pregnancy, was there ever a time when you were pushed, slapped, hit, kicked or beaten by (any of) your husband/partner(s) or anyone else? | | _______No (& No on Q26, Please skip to Q30) _______Yes  _______ Don't know/Don't remember _______ Refused/No answer (Skip to instructions) | | |
| Q27 | During this pregnancy, has your current husband/partner ever forced you to have sexual intercourse when you did not want to, for example by threatening you or holding you down? IF NECESSARY: We define sexual intercourse as vaginal, oral or anal penetration. | | _______No _______Yes  _______ Don't know/Don't remember _______ Refused/No answer (Skip to instructions) | | |
| Q28 | During this pregnancy, did you ever have sexual intercourse you did not want to because you were afraid of what your partner/husband might do if you refused? | | _______No _______Yes  _______ Don't know/Don't remember _______ Refused/No answer (Skip to instructions) | | |
| Q29 | During this pregnancy, did your husband/partner ever force you to do anything else sexual that you did not want or that you found degrading or humiliating? | | _______No _______Yes  _______ Don't know/Don't remember _______ Refused/No answer (Read the instructions) | | |
|  | **If the woman answers: Sometimes/Many times/Most/all the times to Q25 or yes to Q26-29 please [Insert specific local instructions for Health Worker on how to handle case of physical/sexual violence & referral.]** | | | | |
|  | **End of Module 1: Patient History Thank you for answering the questions, we will now move on to the 2nd module of the questionnaire.** | | | | |

|  | **ANC SECTION 2: PATIENT SYMPTOMS** | | | | | | | | |
| --- | --- | --- | --- | --- | --- | --- | --- | --- | --- |
|  | **WHODAS.** *Please read the following: "Now, I would like to ask you some more questions about your everyday activities. This part of the interview is about difficulties people have because of health conditions. (Hand flashcard #1 to respondent) By health condition I mean diseases or illness, or other health problems that may be short or long lasting; injuries; mental or emotional problems; and problems with alcohol or drugs. Remember to keep all of your health problems in mind as you answer the questions.   When I ask you about difficulties in doing an activity think about...(Point to flashcard #1):   - increased effort  - discomfort or pain  - slowness  - changes in the way you do the activity  When answering, I'd like you to think back over the past 30 days. I would also like you to answer these questions thinking about how much difficulty you have had, on average, over the past 30 days, while doing the activity as you usually do it. (Hand flashcard #2 to respondent)   Use this scale when responding. (Read scale aloud): None, mild, moderate, severe, extreme or cannot do. (Ensure that the respondent can easily see flashcards #1 and #2 throughout the interview. Please continue to next question...)"* | | | | | | | | |
|  | **In the past 30 days, how much difficulty did you have in:** | | **None** | **Mild** | **Moderate** | | **Severe** | **Extreme or  cannot do** | |
| Q30 | Standing for long periods such as 30 minutes? | | 1 | 2 | 3 | | 4 | 5 | |
| Q31 | Taking care of your household responsibilities? | | 1 | 2 | 3 | | 4 | 5 | |
| Q32 | Learning a new task, for example, learning how to get to a new place? | | 1 | 2 | 3 | | 4 | 5 | |
| Q33 | How much of a problem did you have joining in community activities (for example, festivities, religious or other activities) in the same way as anyone else can? | | 1 | 2 | 3 | | 4 | 5 | |
| Q34 | How much have you been emotionally affected by your health problems? | | 1 | 2 | 3 | | 4 | 5 | |
|  | **In the past 30 days, how much difficulty did you have in:** | | **None** | **Mild** | **Moderate** | | **Severe** | **Extreme or cannot do** | |
| Q35 | Concentrating on doing something for ten minutes? | | 1 | 2 | 3 | | 4 | 5 | |
| Q36 | Walking a long distance such as a kilometre [or equivalent]? | | 1 | 2 | 3 | | 4 | 5 | |
| Q37 | Washing your whole body? | | 1 | 2 | 3 | | 4 | 5 | |
| Q38 | Getting dressed? | | 1 | 2 | 3 | | 4 | 5 | |
| Q39 | Dealing with people you do not know? | | 1 | 2 | 3 | | 4 | 5 | |
| Q40 | Maintaining a friendship? | | 1 | 2 | 3 | | 4 | 5 | |
| Q41 | Your day-to-day work/school? | | 1 | 2 | 3 | | 4 | 5 | |
| Q42 | Overall, in the past 30 days, how many days were these difficulties present? | | | | | | Record number of days__ | | |
| Q43 | In the past 30 days, for how many days were you totally unable to carry out your usual activities or work because of any health condition? | | | | | | Record number of days__ | | |
| Q44 | In the past 30 days, not counting the days that you were totally unable, for how many days did you cut back or reduce your usual activities or work because of any health condition? | | | | | | Record number of days__ | | |
| Q45 | In the past 30 days, how would you rate your overall health? | | 1 | 2 | 3 | | 4 | | 5 |
|  |  |  | Very Good | Good | Neither poor nor good | | Poor | | Very poor |
|  | **General Symptom(s).**  *Please read the following: "The next few questions I will ask about how you have been feeling, physically, during this pregnancy. Feel free to ask for a break or stop at any time."* | | | | | | | | |
|  | **In the last 30 days, have you experienced any of the following:** *(Please check box if yes, and proceed to next column. If no, skip to next symptom)* | **If yes, how often?** | | | | **If yes, do you still have it today?** | | | |
| Q46 | □ chills | □ most of the time □ occasionally | | | | _______ No ________Yes | | | |
| Q47 | □ nausea | □ most of the time □ occasionally | | | | _______ No ________Yes | | | |
| Q48 | □ fever | □ most of the time □ occasionally | | | | _______ No ________Yes | | | |
| Q49 | □ headache | □ most of the time □ occasionally | | | | _______ No ________Yes | | | |
| Q50 | □ light-headedness | □ most of the time □ occasionally | | | | _______ No ________Yes | | | |
|  |  | | | | | | | | |
| Q51 | □ stiff neck | □ most of the time □ occasionally | | | | _______ No ________Yes | | | |
| Q52 | □ lock jaw | □ most of the time □ occasionally | | | | _______ No ________Yes | | | |
| Q53 | □ sweating profusely/night sweats, unrelated to the heat (diaphoresis) | □ most of the time □ occasionally | | | | _______ No ________Yes | | | |
| Q54 | □ tremor | □ most of the time □ occasionally | | | | _______ No ________Yes | | | |
| Q55 | □ muscle spasms | □ most of the time □ occasionally | | | | _______ No ________Yes | | | |
|  |  | | | | | | | | |
| Q56 | □ chest pain | □ most of the time □ occasionally | | | | _______ No ________Yes | | | |
| Q57 | □ decreased exercise tolerance or fatigue | □ most of the time □ occasionally | | | | _______ No ________Yes | | | |
| Q58 | □ heart beating very fast/too fast (palpitations) | □ most of the time □ occasionally | | | | _______ No ________Yes | | | |
| Q59 | □ seeing stars or spots, blurry vision/flashing lights/floaters (visual disturbance) | □ most of the time □ occasionally | | | | _______ No ________Yes | | | |
| Q60 | □ visual loss | □ most of the time □ occasionally | | | | _______ No ________Yes | | | |
|  |  | | | | | | | | |

| Q61 | □ red/inflamed gums | □ most of the time □ occasionally | | | | _______ No ________Yes | | |
| --- | --- | --- | --- | --- | --- | --- | --- | --- |
| Q62 | □ bleeding gums | □ most of the time □ occasionally | | | | _______ No ________Yes | | |
| Q63 | □ oral lesions | □ most of the time □ occasionally | | | | _______ No ________Yes | | |
| Q64 | □ cough | □ > 2 wks □ < 2 wks | | | | _______ No ________Yes | | |
| Q65 | □ difficulty breathing | □ most of the time □ occasionally | | | | _______ No ________Yes | | |
| Q66 | □ breathing faster than usual | □ most of the time □ occasionally | | | | _______ No ________Yes | | |
|  |  | | | | | | | |
| Q67 | □ vomiting | □ most of the time □ occasionally | | | | _______ No ________Yes | | |
| Q68 | □ vomiting with blood | □ most of the time □ occasionally | | | | _______ No ________Yes | | |
| Q69 | □ abdominal discomfort or pain | □ most of the time □ occasionally | | | | _______ No ________Yes | | |
| Q70 | □ changes in appetite or eating habits | □ most of the time □ occasionally | | | | _______ No ________Yes | | |
|  |  | | | | | | | |
| Q71 | □ pain during urination (dysuria) | □ most of the time □ occasionally | | | | _______ No ________Yes | | |
| Q72 | □ abnormal urination | □ most of the time □ occasionally | | | | _______ No ________Yes | | |
| Q73 | □ changes in bowel habits | □ most of the time □ occasionally | | | | _______ No ________Yes | | |
| Q74 | □ rectal pressure/pain | □ most of the time □ occasionally | | | | _______ No ________Yes | | |
|  |  | | | | | | | |
| Q75 | □ skin rash | □ most of the time □ occasionally | | | | _______ No ________Yes | | |
| Q76 | □ skin lesion | □ most of the time □ occasionally | | | | _______ No ________Yes | | |
| Q77 | □ itching (pruritus) | □ most of the time □ occasionally | | | | _______ No ________Yes | | |
|  |  | | | | | | | |
| Q78 | □ breast tenderness | □ most of the time □ occasionally | | | | _______ No ________Yes | | |
| Q79 | □ feel breast lump (mass) or swelling | □ most of the time □ occasionally | | | | _______ No ________Yes | | |
| Q80 | □ breast redness | □ most of the time □ occasionally | | | | _______ No ________Yes | | |
| Q81 | □ redness in skin of the leg or calf | □ most of the time □ occasionally | | | | _______ No ________Yes | | |
|  |  | | | | | | | |
| Q82 | □ arthralgia/arthritis (joint pain) | □ most of the time □ occasionally | | | | _______ No ________Yes | | |
| Q83 | □ tenderness in leg or calf | □ most of the time □ occasionally | | | | _______ No ________Yes | | |
| Q84 | □ sudden swelling in leg(s) or calf(-ves) | □ most of the time □ occasionally | | | | _______ No ________Yes | | |
| Q85 | □ back pain | □ most of the time □ occasionally | | | | _______ No ________Yes | | |
|  |  | | | | | | | |
| Q86 | □ vaginal bleeding (after sex) | □ most of the time □ occasionally | | | | _______ No ________Yes | | |
| Q87 | □ painful intercourse (dyspareunia) | □ most of the time □ occasionally | | | | _______ No ________Yes | | |
| Q88 | □ pelvic pain | □ most of the time □ occasionally | | | | _______ No ________Yes | | |
| Q89 | □ vaginal discharge (abnormal in color and/or smell) | □ most of the time □ occasionally | | | | _______ No ________Yes | | |
| Q90 | □ spotting or light vaginal bleeding | □ most of the time □ occasionally | | | | _______ No ________Yes | | |
|  |  | | | | | | | |
|  | **In the last 30 days, have you EVER experienced any of the following:** | | | | | | | |
| Q91 | □ urinating blood | _______ No ________Yes | | | | | | |
| Q92 | □ hemorrhoids/piles | _______ No ________Yes | | | | | | |
| Q93 | □ night blindness (difficulty seeing in the dark) | _______ No ________Yes | | | | | | |
| Q94 | □ loss of teeth | _______ No ________Yes | | | | | | |
| Q95 | □ unintentional weight loss | _______ No ________Yes | | | | | | |
| Q96 | □ gained too much weight (excessive weight gain: >1kg per week) | _______ No ________Yes | | | | | | |
| Q97 | □ swollen hands | _______ No ________Yes | | | | | | |
| Q98 | □ stroke | _______ No ________Yes | | | | | | |
| Q99 | □ seizure/fit | _______ No ________Yes | | | | | | |
| Q100 | **Do you know your HIV status?** | _______ No ________Yes (Skip to Q102) | | | | | | |
| Q101 | **Would you like to be tested?** | _______ No ________Yes | | | | | | |
|  | **[Detailed instructions for Health Worker on how to handle case HIV testing referrals.]** | | | | | | | |
| Q102 | **In the last 30 days, have you seen anyone (besides routine pregnancy care) for treatment?** | | | _______No (Skip to Q105) _______Yes | | | | |
| Q103 | **If yes, what did you seek care for?** | | | | | | | |
|  |  | | | | | | | |
| Q104 | **If yes, where did you seek care?** | | | | | | | |
|  |  | | | | | | | |
|  |  | | | | | | | |
| Q105 | **Since you became pregnant, have you been told you have anything wrong/any medical condition?** | | | _______No (Skip to Q107) _______Yes | | | | |
| Q106 | *If yes, please specify:* | | | | | | | |
|  |  | | | | | | | |
|  |  | | | | | | | |
| Q107 | **Are you taking any medications today (including iron, folic acid, Vit A & Vit C, etc)?** | | | _______No (Skip to Q109) _______Yes | | | | |
| Q108 | *If yes, please specify:* | | | | | | | |
|  |  | | | | | | | |
|  |  | | | | | | | |
| Q109 | **Do you have any other medical conditions or problems you would like to report?** | | | _______No (Skip to Q111) _______Yes | | | | |
| Q110 | *If yes, please specify:* | | | | | | | |
|  |  | | | | | | | |
|  |  | | | | | | | |
|  | **Mental Health.** *Please read the following: "The next few questions I will ask about how you have been feeling/your mood during this pregnancy, feel free to ask for a break or stop at any time. I want to remind you that this is confidential and no one will know how you answered. Also, if after this section you'd like to talk more about the questions, I will give you information on where to seek help***."** | | | | | | | |
|  | Over the last 2 weeks, how often have you been bothered by the following problems? | | **Not at all** | | **Several days** | | **More than half the days** | **Nearly every day** |
| Q111 | Feeling nervous, anxious or on edge | | 0 | | 1 | | 2 | 3 |
| Q112 | Not being able to stop or control worrying | | 0 | | 1 | | 2 | 3 |
| Q113 | Worrying too much about different things | | 0 | | 1 | | 2 | 3 |
| Q114 | Trouble relaxing | | 0 | | 1 | | 2 | 3 |
| Q115 | Being so restless that it is hard to sit still | | 0 | | 1 | | 2 | 3 |
| Q116 | Becoming easily annoyed or irritable | | 0 | | 1 | | 2 | 3 |
| Q117 | Feeling afraid as if something awful might happen | | 0 | | 1 | | 2 | 3 |

|  | Over the past 2 weeks, how often have you been bothered by any of the following problems? | **Not at all** | **Several days** | **More than half the days** | **Nearly every day** |
| --- | --- | --- | --- | --- | --- |
| Q118 | Little interest or pleasure in doing things | 0 | 1 | 2 | 3 |
| Q119 | Feeling down, depressed or hopeless | 0 | 1 | 2 | 3 |
| Q120 | Trouble falling asleep, staying asleep or sleeping too much | 0 | 1 | 2 | 3 |
| Q121 | Feeling tired or having little energy | 0 | 1 | 2 | 3 |
| Q122 | Poor appetite or overeating | 0 | 1 | 2 | 3 |
| Q123 | Feeling bad about yourself - or that you're a failure or have let yourself or your family down | 0 | 1 | 2 | 3 |
| Q124 | Trouble concentrating on things, such as reading the newspaper or watching television | 0 | 1 | 2 | 3 |
| Q125 | Moving or speaking so slowly that other people could have noticed. Or, the opposite, being so fidgety or restless that you have been moving around a lot more than usual | 0 | 1 | 2 | 3 |
| Q126 | Thoughts that you would be better off dead or of hurting yourself in some way | 0 | 1 | 2 | 3 |
|  | **Please add up all the points for Q111-Q117. Please add up all the points for Q118-Q126. If total score on EITHER set of questions is equal to 10 or higher, please refer the patients to [insert specific local instructions for Health Worker on how to handle mental health-related case referral.]** | | | | |
|  | **End of Module 2: Symptoms Thank you for answering the questions, we will now move on to the 3rd and final module of the questionnaire, the physical exam.  Do you have any questions?** | | | | |

|  | **ANC SECTION 3: SIGNS/PHYSICAL EXAM** | |
| --- | --- | --- |
|  | **General physical exam.** *At this point in the survey, I will conduct the physical exam. First I will do a general exam, and then check your breasts, your belly, and finally your pelvis and private area (if routine).* | |
| Q127 | Body weight today: | ______ kg |
| Q128 | Height: | ______ cm |
| Q129 | Body temperature (oral or axillary): | ______ °C |
| Q129a | Where on the body was the temperature taken? | □ oral □ axillary  □ other (please specify):__________ |
| Q130 | Pulse rate: | ____/min |
| Q131 | Respiratory rate: | ____/min |
| Q132 | Resting Systolic BP |  |
| Q133 | Resting Diastolic BP |  |
| Q134 | What is the woman's overall health status and appearance? | □ healthy-looking □ ill-looking  □ other:________________ |
| Q135 | What is the woman's affect? | □ normal (full range of emotional expression)  □ blunted (decrease in emotional expression) □ constricted (restricted range of emotional expression) □ flat (absence of affective expression) □ inappropriate (emotions not congruent with context of her thoughts) □ labile (unpredictable shifts in emotional state) □ other:___________________ |
| Q136 | Does she present with conjunctival pallor? | _______No _______Yes (If yes, please specify: □ mild □ moderate □ severe) |
| Q137 | Does she present with jaundiced sclera? | _______No _______Yes |
| Q138 | Does she present with a rash(es)? | _______No _______Yes (If yes, where? Mark with an R in the diagram on Q143) |
| Q139 | Does she present with a lesion(s)? | _______No _______Yes (If yes, where? Mark with an L in the diagram on Q143) |
| Q140 | Does she present with bruises? | _______No _______Yes (If yes, where? Mark with an B in the diagram on Q143) |
| Q141 | Does she present any evidence of self-harm? | _______No _______Yes (If yes, where? Mark with an SH in the diagram on Q143) |
| Q142 | Does she present any evidence of domestic violence? | _______No _______Yes (If yes, where? Mark with an DV in the diagram on Q143) |
| Q143 | When present, please mark the following diagram with the letters corresponding to the questions above:- R for rash(es)- L for lesion(s)- B for bruises- SH for self-harm- DV for domestic violence |  |
| Q144 | Does she present with difficulty in walking/gait abnormality? | _______No _______Yes |
| Q145 | Does she present with goiter/neck swelling? | _______No _______Yes |
| Q146 | Is her thyroid abnormal? | _______No _______Yes |
| Q147 | If yes, please explain: ___________________________________________________________ | |
| Q148 | Are her lymph nodes abnormal? | _______No _______Yes |
| Q149 | If yes, please explain: _____________________________________________________________________ | |
| Q150 | Are her gums bleeding/bloody? | _______No _______Yes |
| Q151 | Are her gums swollen? | _______No _______Yes |
| Q152 | Is her musculoskeletal system abnormal? | _______No _______Yes |
| Q153 | If yes, please explain: ___________________________________________________________ | |
| Q154 | Does she present with any of the following? *(choose all that apply)* | |
|  | □ pitting ankle oedema | □ pitting lower back oedema |
|  | □ oedema of the hands and feet | □ leg swelling |
|  | □ calf tenderness | □ none |
|  | □ other *(please specify)*: __________________________ | |
|  | **Breast exam** | |
| Q155 | Does she present with cracked nipple(s)? | _______No _______Yes |
| Q156 | Does she present with engorged breast(s)? | _______No _______Yes |

| Q157 | Does she present with localized breast tenderness? | _______No _______Yes (If yes, please mark where in the diagram below) |
| --- | --- | --- |
|  |  | **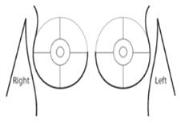** |
| Q158 | Does she present with breast abscess(es)? | _______No _______Yes (If yes, please mark where in the diagram below) |
|  |  | **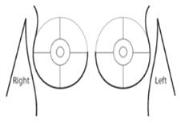** |
| Q159 | Does she present with palpable breast lump(s)? | _______No _______Yes (If yes, please mark where in the diagram below) |
|  |  | 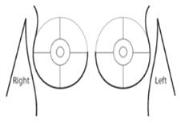 |
|  | **Abdominal exam** | |
| Q160 | Does she present with abdominal tenderness? | _______No _______Yes (If yes, please mark where in the diagram below) |
|  |  | Left  Lower Quadrant  Right Lower Quadrant    Left Upper Quadrant    Right Upper Quadrant |

| Q161 | Does she present with abdominal masses? | _______No _______Yes (If yes, please mark where in the diagram below) |
| --- | --- | --- |
|  |  | Left  Lower Quadrant    Right Lower Quadrant    Left  Upper Quadrant    Right Upper Quadrant |
| Q162 | Does she have a C-section scar? | _______No _______Yes |
|  | **Obstetric exam** | |
| A-2 | Please check symphysis-fundal height | _______ cm |
| A-3 | Please check the fetal heart rate | _____/min |
| A-4 | What is the presentation of the fetus? | □ Cephalic □ Breech  □ Transverse □ >1 fetus |

|  | **Pelvic exam (maybe skipped if not usually part of ANC consultation)** | |
| --- | --- | --- |
| Q163 | Is a vaginal/pelvic exam routine for ANC visit? | |
|  | □ Yes *(please complete this section)* | □ No *(please skip to A-6)* |
|  | □ Yes but only during 1st ANC visit, which patient has already had *(please skip to A-6)* | |
| Q164 | Does she present with any of the following types of FGM? *(please check one)* | □ None |
|  |  | 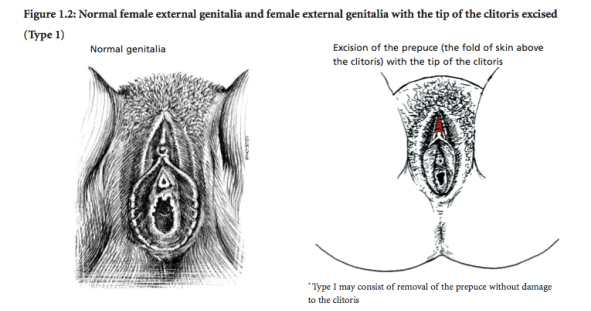□ Type 1: Partial or total removal of the clitoris and/or the prepuce (clitoridectomy) |
|  |  | 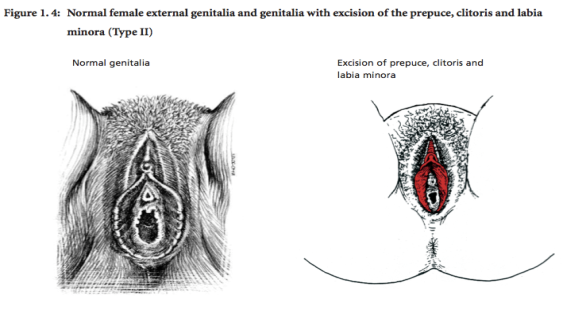□ Type 2: Partial or total removal of the clitoris and the labia minora, with or without excision of the labia majora (excision) |
|  |  | 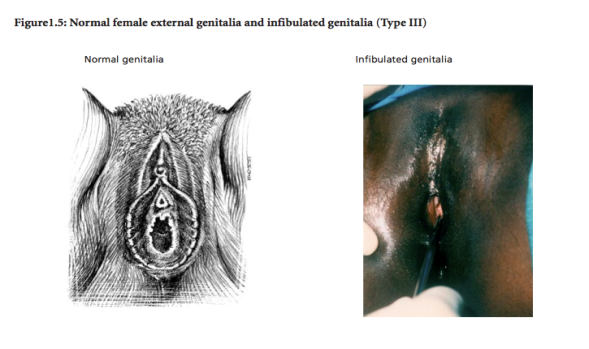□ Type 3: Narrowing of the vaginal orifice with the creation of a covering seal by cutting and appositioning the labia minora and/or the labia majora, with or without excision of the clitoris (infibulation) |
|  |  | 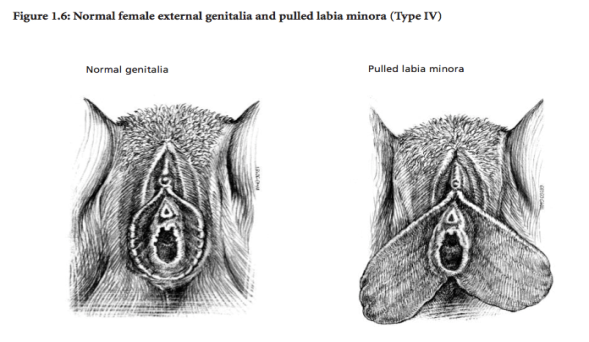□ Type 4: All other harmful procedures to the female genitalia for non-medical purposes, for example: pricking, pulling, piercing, incising, scraping and cauterization |
|  | *Vulva, Vagina & Perineum* | |
| Q165 | Does she present with any of the following in the vulva? (Please check all that apply) | |
|  | □ leakage of urine | □ excoriation |
|  | □ labial swelling | □ none |
|  | □ other (please specify): _______________ | |
| Q166 | Does she present with any of the following in the vagina? (Please check all that apply) | |
|  | □ lesion(s) | □ defects |
|  | □ other (please specify): _____________________ | □ none |
| Q167 | Does she present with any of the following in the perineum? (Please check all that apply) | |
|  | □ excoriation | □ tear |
|  | □ swelling | □ none |
|  | □ other (please specify): __________________ | |
| A-5 | Are there signs of premature membrane rupture? | _______No _______Yes |
| Q168 | Does she present with any abnormal vaginal discharge? | _______No (Skip to QA-6) _______Yes |
| Q169 | If yes, please explain colour: _____________________________________________________________________ | |
|  | **Records & Tests** | |
| A-6 | According to her records, what is the woman's estimated date of delivery (EDD)? (day/month/year) | (dd/mm/yyyy) ____________ □ Don't know |
| A-7 | According to the record, what was the patient's body weight pre-pregnancy? | ______ kg □ not available |
| Q170 | Please identify the source of the patient's records: | |
|  | □ patient health record card (i.e. health passport, mama-baby card, etc.) | □ hospital in-patient record |
|  | □ none available (please ask Q171) | □ other (please specify): ________________ |
| Q171 | If the patient's records are not available, please give reason (select all that apply): | |
|  | □ record does not have necessary information | □ first visit |
|  | □ records cannot be found | □ previous visits at other clinic/center |
|  | □ mother did not bring the mother-baby card | □ records were destroyed |
|  | □ other *(please specify)*: ____________________ | |
|  | **INVESTIGATIONS - Routine Tests.** *Please look through the patient's most up to date medical record to answer the following questions regarding the lab tests she had done.* | |
|  | **Has the patient had any of these tests today?** | |
| Q172 | hemoglobin (hemocue) | _______ No _______Yes  _______ Yes, but results not available If yes, please write in the results: ______ Hb |
| A-8 | syphilis (VDRL) | _______ No _______Yes  _______ Yes, but results not available If yes, please mark one of the results: ____ Reactive _____ Non-reactive _____ Don't know |
| Q173 | nitrite (dipstick) | _______No _______Yes *If yes, please write in the results:*  ________ + ________ ++  ________ +++ _______ None |
| Q174 | leucocytes (dipstick) | _______No _______Yes *If yes, please write in the results:*  ________ + ________ ++  ________ +++ _______ None |
| A-9 | ultrasound (obstetric only - where available) | _______No _______Yes  *If yes, please write in the results:*  _________________________________  _________________________________ |
| Q175 | HIV | _______ No _______Yes _______ Yes, but results not availableIf yes, please mark one of the results:____ Negative _____ Positive _____ Inconclusive |
| Q176 | malaria (RDT or smear) | _______ No _______Yes  _______ Yes, but results not available If yes, please mark one of the results: ____ Negative _____ Positive  _____ Inconclusive |
| Q177 | urine glucose | _______ No _______Yes  _______ Yes, but results not available If yes, please write in the results: ________ + ________ ++  ________ +++ _______ None |
|  | **INVESTIGATIONS - Selective Tests.** *Please look through the patient's most up to date medical record to answer the following questions regarding the lab tests she had done.* | |
| Q178 | glucometer (random blood sugar) | _______ No _______Yes  _______ Yes, but results not available If yes, please write in the results: _________ mMol/L |
|  | **[Include other tests relevant to setting here]** | |
| Q179 | **Has the patient had any other test/exams in the past month?** | _______No _______Yes |
| Q180 | *If yes, please specify the test and results (if available):* | |
|  |  | |
| Q181 | **Have you or the nurse midwife referred the patient for any test/exams to another facility/unit?** | _______No _______Yes  Please ensure referral is in accordance with the SoPs distributed at training. |
| Q182 | *If yes, please specify the test and results (if available):* | |
|  |  | |

| Q183 | **Has the patient had any procedures in the past month?** | _______No _______Yes |
| --- | --- | --- |
| Q184 | *If yes, please specify the procedure and results (if available):* | |
|  |  | |
| Q185 | **Have you or the nurse midwife referred the patient for any procedure to another facility/unit?** | _______No _______Yes  Please ensure referral is in accordance with the SoPs distributed at training. |
| Q186 | *If yes, please specify the procedure and results (if available):* | |
|  |  | |
| Q187 | **Have you or the nurse midwife treated the patient with any medications today?** | _______No _______Yes |
| Q188 | *If yes, please specify the medication:* | |
|  |  | |
| Q189 | **Have you or the nurse midwife referred the patient to obtain/purchase any medication?** | _______No _______Yes |
| Q190 | *If yes, please specify the medication:* | |
|  |  | |
| Q191 | **Did you or the nurse midwife diagnose the patient with any condition(s) today?** | _______No _______Yes |
| Q192 | *If yes, please specify the condition(s):* | |
|  |  | |
| Q193 | **Do you or the nurse midwife have any other comments/notes on the patient?** | _______No _______Yes |
| Q194 | *If yes, please specify:* | |
|  |  | |
|  | **End of Module 3: Patient Signs Thank you for participating in this survey, we have come to the end of the questionnaire.  Do you have any questions for me?** | |
|  |
