# Supplementary material for: Maternal morbidity measurement tool pilot: study protocol
Source: Reprod Health. 2016 Jun 9;13:69. doi: 10.1186/s12978-016-0164-6 (PMC4899915; doi:10.1186/s12978-016-0164-6)
Supplement: Additional file 7: — WHO—ERC Approval. (DOC 563 kb) [file 12978_2016_164_MOESM7_ESM.doc]

**Additional file 7: WHO - ERC Approval**
